# Intensive Care Unit nosocomial sinusitis at the Rasoul Akram Hospital: Tehran, Iran, 2007-2008

**Published:** 2012-09

**Authors:** S Noorbakhsh, M Barati, M Farhadi, J Mousavi, V Zarabi, A Tabatabaei

**Affiliations:** 1 Research Center of Pediatric Infectious Diseases, Rasoul Akram Hospital, Tehran University of Medical Sciences; 2 Research Center for Diseases of Ear, Nose and Throat, Rasoul Akram Hospital, Tehran University of Medical Sciences

**Keywords:** Sinusitis, Nosocomial rhino sinusitis, Nosocomial infection, Intensive Care Units (ICUs)

## Abstract

**Background:**

Nosocomial rhino sinusitis causes major problems in all Intensive Care Units (ICUs).

**Objective:**

To describe incidence, epidemiologic, clinical manifestations, and microbiologic findings in ICUs admitted cases with nosocomial sinusitis.

**Methods& Materials:**

A prospective, cross sectional study done in Pediatric & Adult ICUs in Rasoul Akram Hospital; Tehran Iran (2007-2008). Para-nasal sinus computed tomography (CT) was performed in all adults with fever of unknown origin (FUO) within 48h of admission and repeated thereafter (4-7 days). Infectious sinusitis was diagnosed by microbiological analysis of sinus fluid aspirates.

**Results:**

Acute bacterial nosocomial sinusitis proved in 82% (51/ 63) of all cases. Head trauma was the most common cause; (n = 22, 45%) of cases. The results of culture were positive for 45 cases (82%). Of 45 culture positives, 19 yielded Gram negative organisms (41%) and 9 (22%) gave Gram positives (*S. aureous, Streptococus* spp). The remainders (n = 17, 37%) consisted of mixed aerobic/anaerobic bacteria.

Seven cases, were positive in gram staining of sinus drainage and these were positive in culture for *S. pneumonia* (n = 5)*, Hemophilus influenza* (n = 2). The type of organisms were not related to Glasgow Coma Scale in cases (P = 0.3).

**Conclusion:**

Nosocomial organisms isolated were quite different from community acquired rhino sinusitis cases. Investigation of CT scan and drainage of Para-nasal sinuses would be helpful in undiagnosed FUO cases, especially in traumatic patients. Optimal treatment usually consists of removal of the tubes, mobilizing the patient, and administration the broad-spectrum antibiotics.

## INTRODUCTION

Para nasal sinuses are a common place for infection in children and adults (1, 2). Community acquired rhinosinusitis is one of the most common cause of medical visit by a physician especially a paediatrician (2, 3). Nosocomial infections continues to be a major problem causing morbidity and occasionally mortality in critically ill patients (4). Recently, many authors reported hospital-acquired rhino sinusitis in critically ill patients which present with fever of unknown origin (FUO) (5). Occasionally rhino sinusitis presents with few signs while the patient stay in the intensive care unit (ICU). Nasotracheal or orotracheal intubation, facial trauma, inability to mobilize the patient and prior sinus disease may have role in its occurrence. Diagnosis is usually made with the help of specific radiographs or CT (computed tomography) scans (5, 6). The microbiology of nosocomial rhinosinusitis is quite different from sinusitis in the community (5, 6).

Careful search for nosocomial rhinosinusitis and appropriate treatment is needed to decrease both morbidity, mortality and subsequent other nosocomial infections (7–11). Some authors offer that routine investigation of FUO including sinus CT scan (X ray or ultrasonography), and sinus drainage should be performed if any abnormalities are found. Preventive measures, including the removal of nasogastric tubes in patients requiring long-term mechanical ventilation (12–16)

*Levin et al.,* reported nosocomial rhino sinusitis with imipenem-resistant *Acinetobacter spp* (17). Upper respiratory infection and community acquired rhino sinusitis is common in Iran (18–20). Increasing the antimicrobial resistance reported in Iranian patients (21).

The aim of this study was to determine the incidence, epidemiologic characteristics, clinical manifestations, microbiologic findings and evolution of patients with nosocomial sinusitis in ICUs of Rasoul Akram Hospital in Tehran, Iran.

## MATERIALS AND METHODS

A prospective, cross sectional study was carried out in PICU and ICU in Rasoul Akram Hospital; Tehran Iran (2007-2008). This study was approved by the Ethical Committee in the Research Center of Pediatric Infectious Diseases in Tehran University of Medical Sciences.

Consent letter obtained from patients (or parents). Initially a questionnaire was completed by an authorized physician, followed by complete clinical exams. Para nasal sinus CT was performed only in all adult cases with FUO (but not in children) within 48h of admission in ICU and repeated thereafter ( 4-7 days) if no obvious cause revealed after initial clinical and diagnostic screening (physical examination, microbiological cultures and chest X-ray).

Well-defined nosocomial rhino sinusitis cases selected according to CDC criteria (FUO with no obvious cause and confirmed rhino sinusitis in Para-nasal sinus CT scan).

Phenylephrine drop (0.5%) used by physician in middle meatus of selected cases for antral drainage. Direct smear from the sinus materials were prepared and stained. The specimens were also cultured in both aerobic and anaerobic BACTEC media (Becton Dickenson company) in automated system (BioMerieux). Isolates were identified using the standard techniques (4).

Infectious rhino sinusitis was diagnosed if the specimens were positive in Gram staining (direct smear)/ or positive in rapid antigen detection test (Latex Particle Agglutination). Isolation and identification of organisms from sinus fluid aspirates was also used for diagnosis.

### Cases definition

Patients admitted at ICUs (> 48 hours) who fulfilled criteria of nosocomial rhino sinusitis according to CDC criteria were included in this study (4).

### Exclusion criteria

1) adults cases diagnosed as community acquired rhino sinusitis revealed with abnormality in the first CT scan. Diagnostic parameters for community acquired rhino sinusitis in children based on clinical history/ or imaging diagnostic parameters for rhino sinusitis criteria before admission; 2) Another site for infection concomitant with rhino sinusitis (E.g. meningitis, brain abscess, pneumonia, osteo myelitis, etc).

## RESULTS

**Fig. 1:**
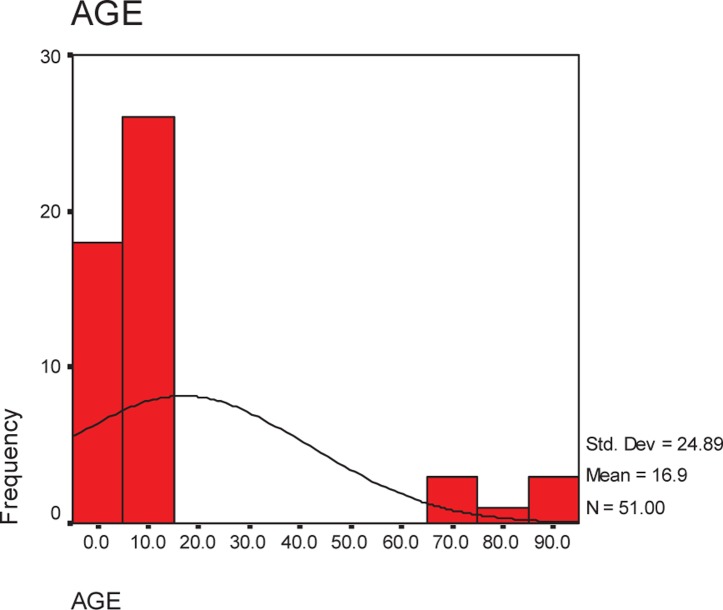
Age distribution in cases of sinusitis.

63 cases had a full CDC criterion and followed for nosocomial rhino sinusitis. 55.8% males, 42.3% females. Cases were between 1-86 years; mean age = 17 + 25 years 58.8% of studied cases (n = 30) aged less than 10 years (Fig. 1).

Head trauma was the most common cause for ICUs admission in 45% (n = 22),other medical diseases (uraemia, aspiration pneumonia, etc): 20.4% (n = 10),brain tumour: 16.3% (n= 8),Cerebral palsy: 2%, face abnormality: 2%.

Glasgow Coma Scale (GCS) < 7 observed in 74.4% (n = 32), 86.5% (n = 32) had NGT (nasogastric tube), 77.8% (n = 43) cases received anti convulsive drugs.

Allergic rhino sinusitis diagnosed in 18% (n = 12) of studied cases. Acute bacterial nosocomial rhino sinusitis proved in 82% (51/63). Positive sinus cultures included Gram negative organisms (*K. pneumoniae, P. aeruginosa* and *Acinetobacter* spp.) in 41% (n = 19) Gram positive organisms (*S. aureus, Streptococcus. spp*) in %22 (n = 9) and mixed aerobic/anaerobic organisms in 37% (n = 17) of cases.

Seven patients (with negative culture) were positive in Gram staining of smear or in rapid antigen detection test (Latex Particle Agglutination) in sinus material included. (*S. pneumoniae* in 5; *H. influenza* in 2 cases). Mean age of cases for Gram negative organisms was seven years; for *S. aureus* 14 years; mixed aerobic/anaerobic infection 27 years. We did not observe any correlation between type of organisms and GCS in cases; (P = 0.3).

## DISCUSSION

Acute bacterial nosocomial rhino sinusitis proved in 82% of studied cases with CDC criteria. Nosocomial sinusitis might be a major problem causing morbidity and mortality in critically ill patients in our center particularly in cases with head trauma (45%). The majority of diagnosed cases (20.4%) had medical diseases (uraemia, aspiration pneumonia, etc. or brain tumour (16.3%). If a careful search for this disease and appropriate treatment are found, decrease in morbidity, mortality and subsequent other nosocomial infections would happen (1, 2). Diagnosis of rhino sinusitis can be made on the basis of a careful history and physical examination. Imaging studies reserved for confirmation of clinical impression or documentation of disease (4–8).

Although fibrotic rhinoscopies used more frequently as an adjunct in adults for the evaluation and management of sinusitis, more studies need to be performed to document its clinical usefulness in children (5, 7).

In present study, 86.5% of cases with nosocomial rhino sinusitis had NGT, which is very close to *Van Zanten et al.* study (7). They reported hospital-acquired sinusitis as a common cause of FUO in orotracheally intubated critically ill patients (7). Indeed, *Arroyo-Sánchez et al* (13) reported fever and endotracheal intubation in all the cases; nasogastric tube in 89%, and purulent rhinorrhea or oral secretions in 83%. In that study, low incidence (1.1%) of nosocomial sinusitis observed in the ICU, but the risk of infectious complications was high (13).

The most common organisms were Gram positive organisms (*S. aureus, Streptococcus* spp) in %22; Gram negative organisms (*K. pneumoniae, P. aeruginosa* and *Acinetobacter* spp) in 41% (n = 19), mixed aerobic/anaerobic organisms in 37% (n = 17) of cases. Similarly, Stein *et al.* described nosocomial sinusitis which frequently isolated *Staphylococcus* spp., *Pseudomonas spp*. and other nosocomial organisms (11). Kriukov *et al.* reported poly bacterial and mono bacterial etiology for nosocomial rhino sinusitis in 70% and 30% respectively (12). The link of late nosocomial pneumonia with nosocomial rhino sinusitis was suggested in 59% patients (14)

*Levin et al.* described severe nosocomial infections with imipenem-resistant Acinetobacter spp; 72.5% of all occurred in the ICU (17). In deed, previous study in our center determined the *Acinetobacter* Infections as one of the nosocomial infections (21).

Previous studies in Tehran showed sinusitis is common in Iranian children. Community acquired rhino sinusitis is one of the most common cause of medical visit by physicians especially paediatricians in our hospital (18–20). Immunologic evaluation in children with sinusitis is necessary, especially in chronic or resistant ones (18).

Nosocomial rhino sinusitis is a well-recognized but insufficiently understood complication of critical illness in patients admitted by ICU in our center. Barati *et al.* explained bacteriological profile and antimicrobial resistance of blood culture isolates, from 456 isolates were obtained from blood cultures, 98 cases had nosocomial infection (21). Here, we observed nosocomial organisms with high antimicrobial resistance like as Barati *et al.* study in our center (21). *Acinetobacter* spp. was the most common agent which isolated from blood cultures of hospital acquired cases (32%), followed by *E. coli* (13.7%) and *Klebsiella* spp. (12%). They did not report any vancomycin-resistant strains of *S. aureus*. Rifampin and ciprofloxacin had good activity against most of Gram-positive and Gram-negative organisms (21). The most effective antibiotics against Gram-negative and Gram-positive bacteria were ciprofloxacin (13% resistance), vancomycin and oxacillin (13% resistance). 43% of *Acinetobacter* spp; 15.4% of *P .aeruginosa* were multi drug resistant (MDR), while 48.7% of *Klebsiella spp* were ESBL-producing isolates. 15% of *S. aureus* were oxacillin-resistant. Carbapenems were highly active against *Enterobacteriaceae* (*E. coli*, *Klebsiell*a) strains with cephalosporines resistance (21)

In conclusion, nosocomial organisms isolated were quite different from community acquired rhino sinusitis cases. Investigation of CT scan and drainage of para-nasal sinuses would be helpful in undiagnosed FUO cases, especially in traumatic patients. Optimal treatment usually consists of removal of the tubes, mobilizing the patient, and administration the broad-spectrum antibiotics.
